# Diagnostic accuracy of alpha-defensin ELISA and lateral flow assays for periprosthetic joint infection: a systematic review and meta-analysis 

**DOI:** 10.5194/jbji-10-525-2025

**Published:** 2025-12-03

**Authors:** Benjamin R. Paul, David G. Deckey, Alex Soriano, Andy Miller, Thorsten M. Seyler

**Affiliations:** 1 Department of Orthopaedic Surgery, Mayo Clinic, Phoenix, Arizona 85054, USA; 2 Creighton University School of Medicine, Phoenix, Arizona 85012, USA; 3 Department of Orthopaedic Surgery, Duke University Medical Center, Durham, North Carolina 27710, USA; 4 Department of Infectious Diseases, Hospital Clinic Barcelona, Barcelona, Spain; 5 Department of Infectious Diseases, Hospital for Special Surgery, New York, New York 10021, USA

## Abstract

**Background**: Alpha-defensin (AD) is a synovial biomarker that can be used in the diagnosis of periprosthetic joint infection (PJI). Two testing modalities are available: the laboratory-based enzyme-linked immunosorbent assay (ELISA) and the point-of-care (POC) lateral flow (LF) assay. Although both assays have been incorporated into modern PJI diagnostic algorithms, their comparative diagnostic accuracy remains incompletely defined. **Methods**: A systematic review and meta-analysis were conducted following Preferred Reporting Items for Systematic reviews and Meta-Analyses (PRISMA) guidelines. PubMed, Embase, and Cochrane databases were searched from 1 January 2000 to 1 February 2024. Studies using contemporary PJI definitions were included. Eligible studies evaluated the diagnostic performance (sensitivity and specificity) of AD-ELISA or AD-LF in patients undergoing evaluation for suspected PJI. Pooled sensitivity and specificity were calculated, and subgroup analyses compared AD assays to traditional synovial markers such as leukocyte count (LC) and polymorphonuclear percentage (PMN %). **Results**: A total of 51 studies met inclusion criteria. Reported sensitivity and specificity varied widely across studies, with median values of 0.86 and 0.97 for AD-ELISA and 0.84 and 0.97 for AD-LF. Pooled estimates, derived from studies reporting confidence intervals, demonstrated a sensitivity and specificity of 87.8 % (95 % CI, 81.2 %–94.3 %) and 97.9 % (95 % CI, 96.5 %–99.2 %) for AD-ELISA and of 81.8 % (95 % CI, 76.0 %–87.5 %) and 97.0 % (95 % CI, 95.9 %–98.2 %) for AD-LF, respectively. Compared with traditional synovial leukocyte count and PMN %, both assays demonstrated comparable or superior specificity, particularly for AD-ELISA. Risk of bias was generally low across included studies. **Conclusion**: Both AD assays demonstrate high specificity in diagnosing PJI, but AD-ELISA offers superior sensitivity compared to AD-LF and traditional synovial markers. Given variability in the underlying diagnostic criteria for PJI, these results should be interpreted within the context of differing reference standards. These findings support the continued use of AD-ELISA as a valid diagnostic modality.

## 1 Introduction

Periprosthetic joint infection (PJI) is a rare but severe complication following total joint arthroplasty (TJA) and is defined by one of several diagnostic criteria commonly used by different organizations, including definitions by the Musculoskeletal Infection Society (MSIS) 2013 (Parvizi and Gehrke, 2014), the International Consensus Meeting (ICM) 2018 (Parvizi et al., 2018), the European Bone and Joint Infection Society (EBJIS) 2021 (McNally et al., 2021), and the Infectious Disease Society of America (IDSA) 2013 (Osmon et al., 2013). These diagnostic criteria are generally a combination of clinical signs, laboratory markers, microbial cultures, histopathologic findings, and intraoperative assessments, with each set of guidelines applying specific thresholds and scoring systems to establish a diagnosis.

Despite advances in diagnostic tools, accurately identifying PJI remains a challenge. No single test offers perfect sensitivity or specificity or has been universally accepted as the diagnostic gold standard. Traditional infection markers, including serum C-reactive protein (CRP), synovial white blood cell count, and intraoperative cultures, are restrained by variable performance, false negatives, and delayed turnaround time (Kheir et al., 2018; Quinlan and Jennings, 2023). Additionally, classic clinical features associated with infection, including fever and leukocytosis, are frequently absent in chronic or low-grade PJI (Slullitel et al., 2018). The diagnostic challenge of diagnosing PJI is further compounded by the significant clinical overlap between clinical presentations of PJI and aseptic failure. Both conditions may present with significant joint pain, swelling, stiffness, and radiographic evidence of implant loosening, making it difficult to differentiate between infectious versus non-infectious etiologies (Patel et al., 2016). This overlap can delay diagnosis and treatment, which ultimately increases the risk for adverse patient outcomes.

In response to these diagnostic challenges, there has been growing interest in the identification of novel biomarkers to improve diagnostic accuracy of PJI detection. Among these, alpha-defensin (AD) has emerged as a promising tool in the diagnostic workup of PJI. AD is an antimicrobial peptide that is released by neutrophils into the synovial fluid upon encountering bacterial pathogens (Lehrer and Ganz, 1992). Unlike conventional markers that may be elevated in the setting of sterile inflammation or mechanical irritation, AD is believed to be more specific to true infection (Deirmengian et al., 2005; Hubert et al., 2024; Lehrer and Ganz, 1992). Two commercially available test formats exist: the laboratory-based alpha-defensin enzyme-linked immunosorbent assay (AD-ELISA) and the point-of-care (POC) alpha-defensin lateral flow (AD-LF) test. Both have demonstrated favorable sensitivity and specificity in prior studies. However, an updated synthesis of the current evidence is necessary to more clearly define and compare the diagnostic performance of these two assays in TJA.

The purpose of this systematic review and meta-analysis was to synthesize the available high-quality evidence on the diagnostic accuracy of both AD-ELISA and AD-LF in the diagnosis of PJI. This study also aimed to explore heterogeneity across the included studies and assess how diagnostic performance varies by test type, study design, and patient population characteristics. Additionally, stratified analyses have compared AD assays with traditional synovial biomarkers such as leukocyte count and polymorphonuclear leukocyte percentage (PMN %), limiting inclusion to studies that excluded these markers from their diagnostic reference standard to reduce incorporation bias.

## 2 Methods

### 2.1 Search strategy

The Preferred Reporting Items for Systematic reviews and Meta-Analyses (PRISMA) methodology was employed (Page et al., 2021). Studies that were published from 1 January 2000 to 1 February 2024 were queried using the following medical databases: PubMed, Embase, and Cochrane. The search strategy employed a combination of the following keywords: “Arthroplasty OR Joint Prosthesis OR Periprosthetic joint infection OR Prosthetic joint infection OR PJI OR Arthroplast OR Total joint OR Joint Prosthe or DAIR or debridement AND synovial fluid OR joint fluid AND Sensitivity and Specificity OR sensitiv OR specificit OR accura OR positive predictive value OR negative predictive value OR PPV OR NPV”. See Appendix A for search criteria specific to each medical database used.

### 2.2 Study assessment and eligibility criteria

The studies included in the meta-analysis were independently selected by two authors. Initial screening involved titles and abstracts, followed by full-text review for studies that met the inclusion criteria or where eligibility was unclear. Any discrepancies regarding study eligibility were resolved by a third author. Included studies met the following criteria: (a) evaluation of AD for diagnosing PJI using either quantitative ELISA or qualitative lateral flow in synovial fluid, (b) clear reporting of sensitivity and specificity, (c) PJI diagnosis based on a recognized reference standard (MSIS 2013, ICM 2018, EBJIS 2021, or IDSA 2013), and (d) publication in English.

### 2.3 Data extraction and quality assessment

The number of PJIs, number of controls, sensitivity, specificity, and confidence intervals were extracted from each study. Quality assessment of each study was conducted using the Quality Assessment of Diagnostic Accuracy Studies (QUADAS) tool (Whiting et al., 2011). The tool evaluates four domains of potential bias: (a) patient selection, (b) index test, (c) reference standard, and (d) flow and timing. Each domain was independently rated as having a low, unclear, or high risk of bias. Discrepancies were resolved through discussion with a third author.

**Figure 1:**
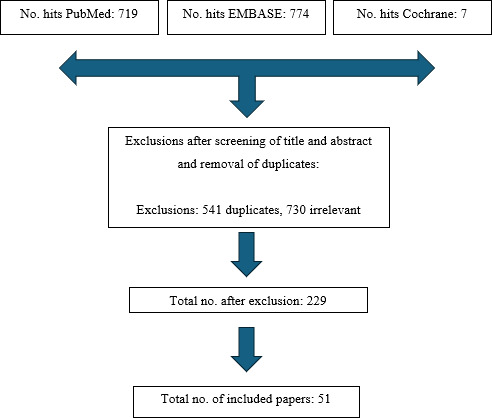
Flow diagram for study selection.

A flow diagram of the study selection is demonstrated in Fig. 1. The initial search yielded 719 records from PubMed, 774 from Embase, and 7 from Cochrane, for a total of 1500 citations from 1 January 2000 to 1 February 2024. After removal of 541 duplicate entries and exclusion of 730 articles based on the abstract and title not meeting the study design criteria, 229 studies remained for full-text review. These exclusions were most commonly due to the article being a review or meta-analysis rather than primary data, commentaries, non-English articles, or studies that did not apply an accepted definition of PJI. After full-text review, 51 studies specific to alpha-defensin met the eligibility criteria and were included in the final analysis.

### 2.4 Statistical analysis

Statistical analysis was performed using Microsoft Excel (Microsoft Corporation, Redmond, Washington) and R software (version 4.4.3; R Core Team, Vienna, Austria). Diagnostic performance data for AD testing (both ELISA and lateral flow assays) were extracted from each study, including sensitivity and specificity. Confidence intervals (95 % CI) were included when provided directly in the study.

Meta-analyses of sensitivity and specificity were performed separately for the AD-ELISA and AD-LF tests. Forest plots were generated using R software. Each subgroup included pooled sensitivity and specificity estimates, accounting for expected heterogeneity among studies. Heterogeneity describes the degree of variability in diagnostic performance estimates (sensitivity and specificity) across studies and was quantified using the I^2^ statistic. Values of 0 % indicate no observed heterogeneity, whereas increasing values reflect greater variability between study results. Only studies that included 95 % CI for sensitivity and specificity were included in the pooled analyses and forest plots. Median values for sensitivity and specificity were calculated across all studies.

## 3 Results

### 3.1 Study selection and characteristics

A total of 51 studies were included in analysis (Table 1). Of these, 22 utilized the quantitative AD-ELISA test, 32 utilized the qualitative AD-LF test, and 2 did not specify the test type. Only 3 studies evaluated both the ELISA and AD-LF tests. In total, 723 patients with PJI were identified in the AD-ELISA group, and 765 patients with PJI were identified in the AD-LF group. Geographically, the studies included in the analysis originated from 15 countries. The majority were conducted in the United States (15) and Germany (10), followed by Italy (4), Austria (4), China (3), the Netherlands (3), Brazil (2), France (3), and Belgium (2); 1 study each came from Taiwan, Japan, Singapore, Türkiye, and Switzerland. Regarding study design, 39 studies were observational clinical studies, 8 studies were prospective, 3 were retrospective, and 1 was a clinical trial.

For the ELISA test, the reported cutoff ranged from 4.8 to 35.4 µg mL^−1^, although 5 studies did not disclose a specific threshold. In contrast, the AD-LF test is a qualitative test and therefore does not involve a defined numerical cutoff (Table 1).

**Table 1:** Summary of articles included in the analysis.^1^

Author (year) (reference)	Index test (detection method, cutoff)	Reference standard	Country	Study type
Grünwald (2023) (Grünwald et al., 2023)	AD (ELISA, –)	EBJIS 2021	Germany	Observational Clinical Study (OCS)
Li (2023) (Li et al., 2023)	AD (ELISA, –)	MSIS 2013	China	OCS
Abdo (2022) (Abdo et al., 2022)	AD (ELISA, –)	MSIS 2013	Brazil	OCS
Kuo (2022) (Kuo et al., 2022)	AD (ELISA,5.2 mg L^−1^)	ICM 2018	Taiwan	OCS
Abdo (2021) (Abdo et al., 2021)	AD (ELISA, 5.2 mg L^−1^)	MSIS 2013	Brazil	OCS
Levent (2021) (Levent et al., 2021)	AD (ELISA, 5.2 mg L^−1^)	ICM 2018	Germany	OCS
Shohat (2021) (Shohat et al., 2021)	AD (ELISA, 5.2 mg L^−1^)^2^	ICM 2018	USA	OCS
Ivy (2021) (Ivy et al., 2021)	AD (ELISA, 5.2 mg L^−1^)^2^	MSIS 2013	USA	OCS
Yu (2021) (Yu et al., 2021)	AD (ELISA, 7.15 µg mL^−1^)	Other^3^	China	Retrospective
Li (2021) (Li et al., 2021)	AD (ELISA, 35.4 µg mL)	MSIS 2013	China	OCS
Busch (2020) (Busch et al., 2020)	AD (ELISA, 4800 ng mL^−1^)	ICM 2018	Germany	OCS
Kleiss (2019) (Kleiss et al., 2019)	AD (ELISA, 5.2 mg L^−1^)^2^	MSIS 2013	Germany	OCS
Ettinger (2019) (Ettinger et al., 2020)	AD (ELISA)	MSIS 2013	Germany	OCS
Miyamae (2019) (Miyamae et al., 2019)	AD (ELISA, 5.2 mg L^−1^)^2^	MSIS 2013	USA	OCS
De Vecchi (2018) (De Vecchi et al., 2018)	AD (ELISA, 5.2 mg L^−1^)^2^	ICM 2014	Italy	OCS
		EBJIS 2021^4^		
Sigmund (2018) (Sigmund et al., 2018)	AD (ELISA, 5.2 mg L^−1^)^2^	MSIS 2013	Germany	Retrospective
		IDSA 2013		
Kelly (2018) (Kelly et al., 2018)	AD (ELISA, 5.2 mg L^−1^)^2^	MSIS 2013	USA	OCS
Kanwar (2018) (Kanwar et al., 2018)	AD (ELISA, 5.2 mg L^−1^)^2^	MSIS 2013	USA	OCS
Okroj (2018) (Okroj et al., 2018)	AD (ELISA)	MSIS 2013	USA	OCS
Deirmengian (2014) (Deirmengian et al., 2014a)	AD (ELISA, 5.2 mg L^−1^)^2^	MSIS 2013	USA	OCS
Deirmengian (2015) (Deirmengian et al., 2015a)	AD (ELISA, 5.2 mg L^−1^)^2^	MSIS 2013	USA	OCS
Deirmengian (2014) (Deirmengian et al., 2014b)	AD (ELISA, 5.2 mg L^−1^)^2^	MSIS 2013	USA	OCS
Deirmengian (2021) (Deirmengian et al., 2021)	AD (LF)	MSIS 2013	USA	Clinical trial
		MSIS 2013		
Kuiper (2022) (Kuiper et al., 2022)	AD (LF)	ICM 2018^5^	Netherlands	OCS
		EBJIS 2021^5^		
Baker (2022) (Baker et al., 2022)	AD (LF)	ICM 2018	USA	OCS
Mori (2022) (Mori et al., 2022)	AD (LF)	MSIS 2013	Japan	OCS
Abdo (2021) (Abdo et al., 2021)	AD (LF)	MSIS 2013	Brazil	OCS
Ivy (2021) (Ivy et al., 2021)	AD (LF)	MSIS 2013	USA	OCS
Obiang (2020) (Obiang et al., 2020)	AD (LF)	MSIS 2013	Belgium	OCS
Chisari (2021) (Chisari et al., 2021)	AD (LF)	ICM 2018	USA	OCS

**Table 1:** Continued.

Author (year) (reference)	Index test (detection method, cutoff)	Reference standard	Country	Study type
Petrucca (2020) (Petrucca et al., 2020)	AD (LF)	MSIS 2013	Italy	OCS
Weigelt (2020) (Weigelt et al., 2020)	AD (LF)	MSIS 2013	Austria	Prospective
		MSIS 2013		
Huard (2020) (Huard et al., 2020)	AD (LF)	ICM 2018	France	OCS
		IDSA 2013		
		EBJIS 2021		
Ding (2019) (Ding et al., 2019)	AD (LF)	MSIS 2013	Singapore	OCS
Tahta (2019) (Tahta et al., 2019)	AD (LF)	MSIS 2013	Türkiye	Prospective
Riccio (2018) (Riccio et al., 2018)	AD (LF)	MSIS 2013	Italy	Prospective
		MSIS 2013		
Sigmund (2018) (Sigmund et al., 2018)	AD (LF)	IDSA 2013	Germany	Retrospective
		EBJIS 2021^5^		
		Other^3^		
Renz (2018) (Renz et al., 2018)	AD (LF)	IDSA 2013	Germany	Prospective
		MSIS 2013		
De Saint Vincent (2018) (de Saint Vincent et al., 2018)	AD (LF)	MSIS 2013	France	OCS
Plate (2018) (Plate et al., 2018)	AD (LF)	MSIS 2013	Switzerland	OCS
Gehrke (2018) (Gehrke et al., 2018)	AD (LF)	MSIS 2013	Germany	OCS
Balato (2018) (Balato et al., 2018)	AD (LF)	ICM 2018	Italy	OCS
Berger (2017) (Berger et al., 2017)	AD (LF)	MSIS 2013	Belgium	Prospective
Suda (2017) (Suda et al., 2017)	AD (LF)	MSIS 2013	Germany	Prospective
Sigmund (2017) (Sigmund et al., 2017)	AD (LF)	Modified MSIS 2013^6^	Austria	Prospective
Kasparek (2016) (Kasparek et al., 2016)	AD (LF)	MSIS 2013	Austria	OCS
De Saint Vincent (2021) (de Saint Vincent et al., 2021)	AD (LF)	ICM 2018	France	OCS
Sigmund (2019) (Sigmund et al., 2019)	AD (LF)	MSIS 2013	Austria	Prospective
		EBJIS 2021		
Kuiper (2020) (Kuiper et al., 2020a)	AD (LF)	MSIS 2013	Netherlands	OCS
		ICM 2018		
Scholten (2018) (Scholten et al., 2018)	AD (LF)	Positive intraoperative culture	Netherlands	OCS
Bonanzinga (2017) (Bonanzinga et al., 2017)	AD (LF)	ICM 2014	Germany	OCS
Frangiamore (2016) (Frangiamore et al., 2016)	AD (LF)	MSIS 2013	USA	OCS
Bingham (2014) (Bingham et al., 2014)	AD (not specified)	MSIS 2013	USA	OCS
Stone (2018) (Stone et al., 2018)	AD (not specified)	MSIS 2013	USA	Retrospective

^1^ For reporting the median sensitivity and specificity in the summary report, in those studies where different criteria were evaluated, we used the results for the most recent criteria. ^2^ Providing results as a semiquantitative signal-to-cutoff ratio of 1.0. ^3^ Criteria: (1) purulence around the prosthesis or sinus tract, (2) increased synovial fluid leukocyte count, (3) positive histopathology, or (4) significant microbial growth in the synovial fluid, periprosthetic tissue, or sonicated culture. ^4^ Corresponds to the working draft that was finally published as EBJIS 2021 criteria. ^5^ Regarding inconclusive (ICM 2018) and likely (EBJIS 2021) as infected cases. ^6^ One of the following major criteria was present: a sinus track communicating with the joint, two positive conventional cultures with phenotypically identical microorganisms, or three minor criteria being met (elevated serum CRP levels, one positive microbiological culture, and positive histological analysis of periprosthetic tissue).

### 3.2 Diagnostic accuracy

#### 3.2.1 Alpha-defensin ELISA assay

A total of 22 studies evaluated the diagnostic accuracy of the AD-ELISA test for the diagnosis of PJI, reporting a wide range of sensitivities and specificities (Table 2). Sensitivity values range from 0.5 (Sigmund et al., 2018) to 1.00 (Okroj et al., 2018; Deirmengian et al., 2015a, 2014b). Specificity ranged from 0.68 (Okroj et al., 2018) to 1.00 in multiple studies (Abdo et al., 2021; Deirmengian et al., 2014a, b, 2015b; Li et al., 2023, 2021; Miyamae et al., 2019). The median sensitivity and specificity were 0.8635 and 0.9675, respectively. When stratified by reference standard, the median sensitivity and specificity were 0.685 and 0.985 for EBJIS 2021, 0.927 and 0.963 for MSIS 2013, and 0.844 and 0.931 for ICM 2018.

**Table 2:** Sensitivity and specificity of the alpha-defensin ELISA test for the diagnosis of periprosthetic joint infection across included studies, stratified by reference standard^1^.

Author (year) (reference)	Index test	Reference	PJI	Controls	Sensitivity	Specificity		
		standard	( n )	( n )	(95 % CI)	(95 % CI)		
Grünwald (2023) (Grünwald et al., 2023)	AD (ELISA, –)	EBJIS 2021	54	195	0.87, –	0.99, –		
Sigmund (2018) (Sigmund et al., 2018)	AD (ELISA, 5.2 mg L^−1^)	EBJIS 2021	22	49	0.5 (0.31–0.69)	0.98 (0.88–1)		
Median	–	EBJIS 2021	–	–	0.685	0.985		
Li (2023) (Li et al., 2023)	AD (ELISA, –)	MSIS 2013	48	26	0.78 (0.64–0.87)	1 (0.0815–1)		
Abdo (2022) (Abdo et al., 2022)	AD (ELISA, –)	MSIS 2013	17	23	0.941 (0.73–0.99)	1 (0.86–1)		
Abdo (2021) (Abdo et al., 2021)	AD (ELISA, 5.2 mg L^−1^)	MSIS 2013	22	31	0.955 (0.772–0.999)	1 (0.888–1)		
Ivy (2021) (Ivy et al., 2021, p. 20)	AD (ELISA, 5.2 mg L^−1^)	MSIS 2013	18	81	0.833 (0.586–0.964)	0.963 (0.897–0.992)		
Li (2021)	AD (ELISA, 35.4 µg mL^−1^)	MSIS 2013	25	25	0.96, –	1, –		
Kleiss (2019) (Kleiss et al., 2019)	AD (ELISA, 5.2 mg L^−1^)	MSIS 2013	55	150	0.782 (0.667–0.885)	0.966 (0.93–0.993)		
Ettinger (2019) (Ettinger et al., 2020)	AD (ELISA)	MSIS 2013	12	60	0.75, –	0.883, –		
Miyamae (2019) (Miyamae et al., 2019)	AD (ELISA, 5.2 mg L^−1^)	MSIS 2013	15	36	0.93, –	1, –		
Sigmund (2018) (Sigmund et al., 2018)	AD (ELISA, 5.2 mg L^−1^)	MSIS 2013	22	49	0.85 (0.56–0.97)	0.98 (0.9–1)		
Kelly (2018) (Kelly et al., 2018)	AD (ELISA, 5.2 mg L^−1^)	MSIS 2013	11	28	0.82, –	0.82, –		
Kanwar (2018) (Kanwar et al., 2018)	AD (ELISA, 5.2 mg L^−1^)	MSIS 2013	35	35	0.971 (0.92–1.03)	0.971 (0.92–1.03)		
Okroj (2018) (Okroj et al., 2018)	AD (ELISA)	MSIS 2013	1	25	1, –	0.68, –		
Deirmengian (2014) (Deirmengian et al., 2014a)	AD (ELISA, 5.2 mg L^−1^)	MSIS 2013	37	112	0.973 (0.858–0.996)	0.955 (0.899–0.985)		
Deirmengian (2015)	AD (ELISA, 5.2 mg L^−1^)	MSIS 2013	23	23	1 (1–1)	1 (1–1)		
Deirmengian (2014)	AD (ELISA, 5.2 mg L^−1^)	MSIS 2013	29	66	1 (0.88–1)	1 (0.95–1)		
Median	–	MSIS 2013	–	–	0.927	0.963		
Kuo (2022) (Kuo et al., 2022)	AD (ELISA, 5.2 mg L^−1^)	ICM 2018	42	34	0.83, –	0.94, –		
Levent (2021) (Levent et al., 2021)	AD (ELISA, 5.2 mg L^−1^)	ICM 2018	109	151	0.917, –	0.921, –		
Shohat (2021) (Shohat et al., 2021)	AD (ELISA, 5.2 mg L^−1^)	ICM 2018	28	94	0.857 (0.673–0.96)	0.969 (0.913–0.994)		
Busch (2020) (Busch et al., 2020)	AD (ELISA, 4800 ng mL^−1^)	ICM 2018	23	47	0.52, –	0.88, –		
Median	–	ICM 2018	–	–	0.844	0.931		
De Vecchi (2018) (De Vecchi et al., 2018)	AD (ELISA, 5.2 mg L^−1^)	ICM 2014	32	34	0.844 (0.665–0.941)	0.941 (0.789–0.989)		
Sigmund (2018) (Sigmund et al., 2018)	AD (ELISA, 5.2 mg L^−1^)	IDSA 2013	22	49	0.73 (0.48–0.89)	0.98 (0.88–1)		
Yu (2021) (Yu et al., 2021)	AD (ELISA, 7.15 µg mL^−1^)	Other^2^	65	65	0.831, –	0.857, –		
Overall median	–	All definitions	–	–	0.8635	0.9675		

^1^ For reporting the median sensitivity and specificity in the summary report, in those studies where different criteria were evaluated, we used the results for the most recent criteria. ^2^ Criteria: (1) purulence around the prosthesis or sinus tract, (2) increased synovial fluid leukocyte count, (3) positive histopathology, or (4) significant microbial growth in the synovial fluid, periprosthetic tissue, or sonicated culture.

**Figure 2:**
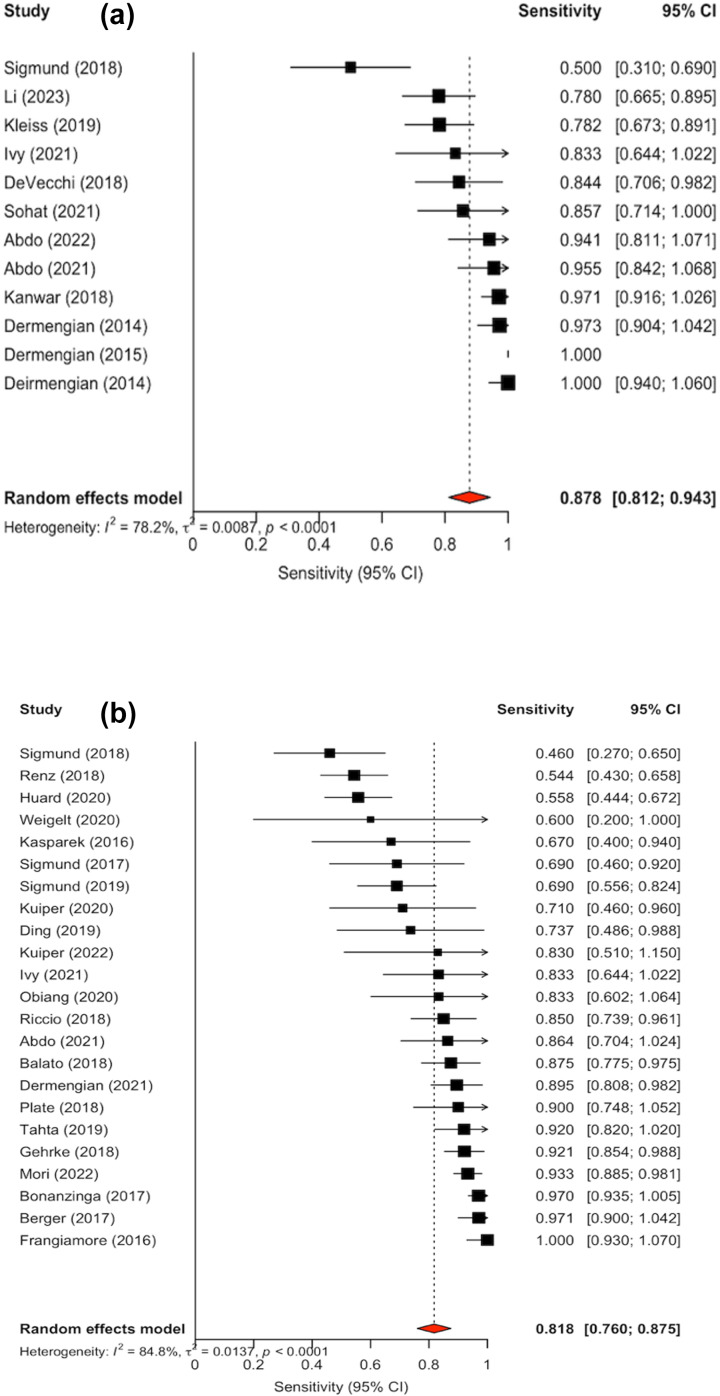
**(a)** Forest plot of sensitivity for alpha-defensin ELISA assay in diagnosing periprosthetic joint infection. **(b)** Forest plot of sensitivity for alpha-defensin lateral flow assay in diagnosing periprosthetic joint infection.

**Figure 3:**
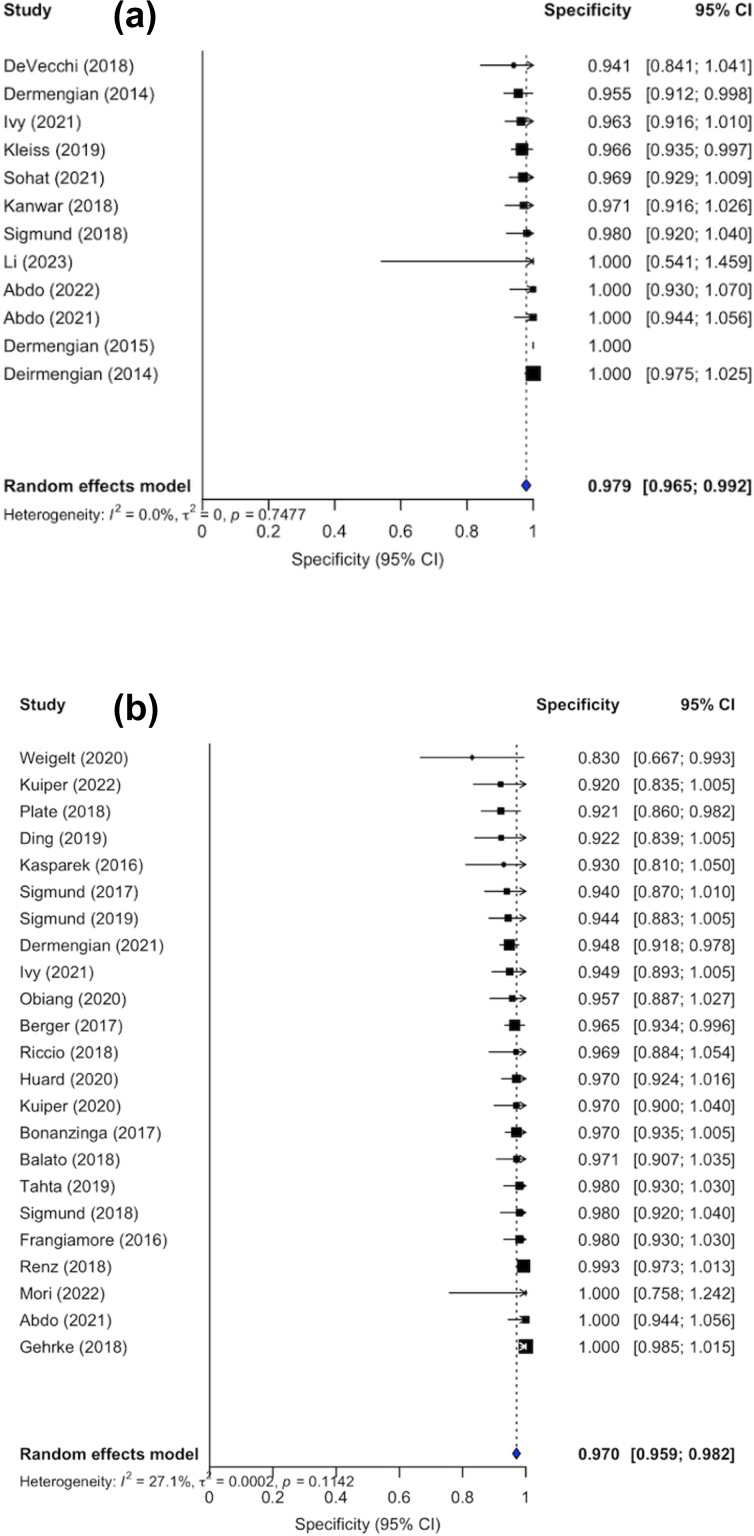
**(a)** Forest plot of specificity for alpha-defensin ELISA assay in diagnosing periprosthetic joint infection. **(b)** Forest plot of specificity for alpha-defensin lateral flow assay in diagnosing periprosthetic joint infection.

Confidence intervals were inconsistently reported; 10 out of the 22 studies did not include confidence interval in their analysis. Forest plots illustrating sensitivity (Fig. 2a) and specificity (Fig. 3a) were created exclusively from studies that included confidence interval data. The pooled sensitivity and specificity of the AD-ELISA assay was 0.878 (95 % CI: 0.812 to 0.943) and 0.979 (95 % CI: 0.965 to 0.992), respectively. Heterogeneity among studies for AD-ELISA assay was 78.2 % for sensitivity and 0 % for specificity.

#### 3.2.2 Alpha-defensin lateral flow assay

The diagnostic accuracy of the AD-LF assay was evaluated across 30 studies (Table 3). Sensitivity and specificity values varied across studies, ranging from 0.2 (Scholten et al., 2018) to 1.00 (Frangiamore et al., 2016) and 0.824 (Suda et al., 2017) to 1.0, respectively (Abdo et al., 2021; Mori et al., 2022; Chisari et al., 2021; Gehrke et al., 2018; Scholten et al., 2018). The median sensitivity and specificity of the AD-LF was 0.8415 and 0.967, respectively. When stratified by reference standard, the median sensitivity and specificity were 0.514 and 0.975 for EBJIS 2021, 0.869 and 0.958 for MSIS 2013, 0.878 and 0.971 for ICM 2018, 0.673 and 0.955 for IDSA 2013, and 0.372 and 0.997 for studies using other criteria.

Confidence intervals were reported in the analysis of 23 out of the 30 studies. Forest plots for sensitivity (Fig. 2b) and specificity (Fig. 3b) were generated from the 23 studies with reported confidence intervals. The pooled sensitivity and specificity of the AD-LF assay were 0.818 (95 % CI: 0.760 to 0.875) and 0.970 (95 % CI: 0.959 to 0.982), respectively. Heterogeneity among studies for the AD-LF assay was 84.8 % for sensitivity and 27.1 % for specificity.

**Table 3:** Sensitivity and specificity of alpha-defensin lateral flow test for the diagnosis of periprosthetic joint infection across included studies, stratified by reference standard.^*^

Author (year) (reference)	Index test	Reference	PJI	Controls	Sensitivity	Specificity
		standard	( n )	( n )	(95 % CI)	(95 % CI)
Kuiper (2022) (Kuiper et al., 2022)	AD (LF)	EBJIS 2021	20	37	0.47 (0.24–1)	1 (0.91–1)
Huard (2020) (Huard et al., 2020)	AD (LF)	EBJIS 2021	68	68	0.558 (0.441–0.67)	0.97 (0.899–0.992)
Sigmund (2018) (Sigmund et al., 2018)	AD (LF)	EBJIS 2021	22	49	0.46 (0.27–0.65)	0.98 (0.88–1)
Kuiper (2020) (Kuiper et al., 2020a)	AD (LF)	EBJIS 2021	14	38	0.71 (0.42–0.92)	0.97 (0.86–1)
Median	–	EBJIS 2021	–	–	0.514	0.975
Deirmengian (2021)^61^	AD (LF)	MSIS 2013	57	248	0.895 (0.785–0.96)	0.948 (0.912–0.972)
Mori (2022) (Mori et al., 2022)	AD (LF)	MSIS 2013	15	3	0.933 (0.837–0.933)	1 (0.516–1)
Abdo (2021) (Abdo et al., 2021)	AD (LF)	MSIS 2013	22	31	0.864 (0.651–0.971)	1 (0.888–1)
Ivy (2021) (Ivy et al., 2021)	AD (LF)	MSIS 2013	16	81	0.833 (0.586–0.964)	0.949 (0.874–0.986)
Obiang (2020) (Obiang et al., 2020)	AD (LF)	MSIS 2013	8	52	0.833 (0.516–0.979)	0.957 (0.855–0.995)
Petrucca (2020) (Petrucca et al., 2020)	AD (LF)	MSIS 2013	8	10	0.875, –	0.9, –
Weigelt (2020) (Weigelt et al., 2020)	AD (LF)	MSIS 2013	5	24	0.6 (0.146–0.947)	0.83 (0.626–0.953)
Huard (2020) (Huard et al., 2020)	AD (LF)	MSIS 2013	41	95	0.878 (0.744–0.946)	0.959 (0.894–0.983)
Ding (2019) (Ding et al., 2019)	AD (LF)	MSIS 2013	19	51	0.737 (0.488–0.99)	0.922 (0.811–0.978)
Tahta (2019) (Tahta et al., 2019)	AD (LF)	MSIS 2013	17	21	0.92 (0.8–1)	0.98 (0.9–1)
Riccio (2018) (Riccio et al., 2018)	AD (LF)	MSIS 2013	40	33	0.85 (0.72–0.943)	0.969 (0.828–0.999)
Sigmund (2018) (Sigmund et al., 2018)	AD (LF)	MSIS 2013	13	58	0.77 (0.49–0.92)	0.98 (0.9–1)
Renz (2018) (Renz et al., 2018)	AD (LF)	MSIS 2013	45	167	0.844 (0.705–0.935)	0.964 (0.923–0.987)
De Saint Vincent (2018) (de Saint Vincent et al., 2018)	AD (LF)	MSIS 2013	9	33	0.889, –	0.96, –
Plate (2018) (Plate et al., 2018)	AD (LF)	MSIS 2013	20	89	0.9 (0.683–0.988)	0.921 (0.845–0.968)
Gehrke (2018) (Gehrke et al., 2018)	AD (LF)	MSIS 2013	76	119	0.921 (0.836–0.971)	1 (0.97–1)
Berger (2017) (Berger et al., 2017)	AD (LF)	MSIS 2013	34	87	0.971 (0.857–0.999)	0.965 (0.93–0.992)
Suda (2017) (Suda et al., 2017)	AD (LF)	MSIS 2013	13	17	0.769, –	0.824, –
Kasparek (2016) (Kasparek et al., 2016)	AD (LF)	MSIS 2013	12	28	0.67 (0.35–0.89)	0.93 (0.75–0.99)
Sigmund (2019) (Sigmund et al., 2019)	AD (LF)	MSIS 2013	29	72	0.69 (0.56–0.828)	0.944 (0.86–0.982)
Kuiper (2020) (Kuiper et al., 2020a)	AD (LF)	MSIS 2013	6	46	1 (0.54–1)	0.89 (0.76–0.96)
Frangiamore (2016) (Frangiamore et al., 2016)	AD (LF)	MSIS 2013	24	54	1 (0.86–1)	0.98 (0.9–1)
Median	–	MSIS 2013	–	–	0.8695	0.958
Sigmund (2017) (Sigmund et al., 2017)	AD (LF)	Modified MSIS	13	36	0.69 (0.46–0.92)	0.93 (0.75–0.99)
Kuiper (2022) (Kuiper et al., 2022)	AD (LF)	ICM 2018	17	40	0.86 (0.42–1)	1 (0.91–1)
Baker (2022) (Baker et al., 2022)	AD (LF)	ICM 2018	194	394	0.986, –	0.869, –
Chisari (2021) (Chisari et al., 2021)	AD (LF)	ICM 2018	78	181	0.792, –	1, –
Huard (2020) (Huard et al., 2020)	AD (LF)	ICM 2018	41	95	0.878 (0.744–0.946)	0.957 (0.896–0.983)
Balato (2018) (Balato et al., 2018)	AD (LF)	ICM 2018	16	35	0.875 (0.746–0.947)	0.971 (0.869–0.997)
De Saint Vincent (2021) (de Saint Vincent et al., 2021)	AD (LF)	ICM 2018	22	90	0.955, –	0.91, –
Kuiper (2020) (Kuiper et al., 2020a)	AD (LF)	ICM 2018	10	40	0.91 (0.62–1)	1 (0.91–1)
Median	–	ICM 2018	–	–	0.878	0.971
Bonanzinga (2017) (Bonanzinga et al., 2017)	AD (LF)	ICM 2014	33	123	0.97 (0.92–0.99)	0.97 (0.92–0.99)
Huard (2020) (Huard et al., 2020)	AD (LF)	IDSA 2013	50	86	0.7 (0.562–0.809)	0.942 (0.871–0.975)
Sigmund (2018) (Sigmund et al., 2018)	AD (LF)	IDSA 2013	15	56	0.67 (0.42–0.85)	0.98 (0.9–1)
Renz (2018) (Renz et al., 2018)	AD (LF)	IDSA 2013	55	157	0.673 (0.533–0.793)	0.955 (0.91–0.982)
Median	–	IDSA 2013	–	–	0.673	0.955
Renz (2018) (Renz et al., 2018)	AD (LF)	Other	79	133	0.544 (0.428–0.657)	0.993 (0.959–1)
Scholten (2018) (Scholten et al., 2018)	AD (LF)	Positive culture	5	32	0.2, –	1, –
Median	–	Other	–	–	0.372	0.9965
Overall median		All definitions			0.8415	0.967

^*^ For reporting the median sensitivity and specificity in the summary report, in those studies where different criteria were evaluated, we used the results for the most recent criteria.

#### 3.2.3 Synovial fluid biomarkers (SFBs)

Synovial fluid biomarkers (SFBs) reflect the presence or activity of neutrophils. This analysis evaluated studies that compared the diagnostic performance of SFBs (AD-ELISA and AD-LF) against the standard synovial fluid tests of leukocyte count (LC) and percentage of polymorphonuclear neutrophils (PMN %). To prevent incorporation bias, only studies that excluded LC and PMN % from their PJI diagnostic criteria were included in the sub-analysis.

#### 3.2.4 AD-ELISA

A total of 6 studies met the criteria for the AD-ELISA cohort comparison. The median sensitivity of AD-ELISA, LC, and PMN was 0.844 (95 % CI: 0.78 to 0.93), 0.872 (95 % CI: 0.74 to 0.93), and 0.881 (95 % CI: 0.796 to 0.94), respectively. The median specificity of AD-ELISA, LC, and PMN was 0.941 (95 % CI: 0.921 to 1), 0.912 (95 % CI: 0.71 to 1), and 0.84 (95 % CI: 0.682 to 0.841), respectively (Table 4).

**Table 4:** Comparison of AD-ELISA, synovial fluid leukocyte count, and PMN % for PJI diagnosis.

Author (year) (reference)	S of AD-ELISA	S of LC (cutoff in cells µL ^−1^)	S of PMN (cutoff in %)	Sp of AD-ELISA	Sp of LC	Sp of PMN
Li (2023) (Li et al., 2023)	0.78	0.74 (3000)	0.796 (70)	1	1	0.682
Kuo (2022) (Kuo et al., 2022)	0.83	0.68 (3000)	0.94 (70)	0.94	0.71	0.84
Levent (2021) (Levent et al., 2021)	0.917	0.872 (3000)	0.881 (70)	0.921	0.899	0.841
Ivy (2021) (Ivy et al., 2021)	0.833	0.833^1^	0.899^1^	0.963	0.808	0.872
Miyamae (2019) (Miyamae et al., 2019)	0.93	0.93 (3000)	–	1	1	–
De Vecchi (2018) (De Vecchi et al., 2018)	0.844	0.937 (3000)	–	0.941	0.912	–
Median (range)^2^	0.844 (0.78–0.93)	0.872 (0.74–0.93)	0.881 (0.796–0.94)	0.941 (0.921–1)	0.912 (0.71–1)	0.84 (0.682–0.841)

S, sensitivity. Sp, specificity. AD-LF, alpha-defensin lateral flow. LC, leukocyte count. PMN, polymorphonuclear neutrophils. %, percentage. ^1^ The cutoff for the LC was 3000 cells µ L^−1^ in total hip arthroplasties (THAs) and 1700 cells µ L^−1^ in total knee arthroplasties (TKAs). The cutoff for PMN was 80 % in THAs and 65 % in TKAs. ^2^ Considering only articles using an LC cutoff of 3000 cells µ L^−1^ and a PMN of 70 %. Consideration: using an LC cutoff of 3000 cells µ L^−1^ and a PMN of 70 %, AD-ELISA showed similar sensitivity and slightly higher specificity compared to both LC and PMN %.

#### 3.2.5 AD-LF

A total of 11 studies met the criteria for the AD-LF cohort. The median sensitivity of AD-LF, LC, and PMN was 0.85 (95 % CI: 0.52 to 0.971), 0.864 (95 % CI: 0.75 to 0.902), and 0.842 (95 % CI: 0.75 to 0.895), respectively. The median specificity of AD-LF, LC, and PMN was 0.969 (95 % CI: 0.922 to 1), 0.94 (5 % CI: 0.764 to 0.989), and 0.949 (95 % CI: 0.903 to 1), respectively (Table 5).

**Table 5:** Comparison of AD-LF, synovial fluid leukocyte count, and PMN % for PJI diagnosis.

Author (year)	S of AD-LF	S of LC (cutoff in cells µ L^−1^)	S of PMN (cutoff in %)	Sp of AD-LF	Sp of LC	Sp of PMN
Deirmengian (2021)	0.941	0.902 (3000)	0.784 (80)	0.944	0.927	0.977
Ivy (2021) (Ivy et al., 2021)	0.833	0.833^1^	0.8991	0.949	0.808	0.872
Chisari (2021) (Chisari et al., 2021)	0.792	0.872 (3000)	0.895 (80)	1	0.989	0.928
Ding (2019) (Ding et al., 2019)	0.737	0.89 (3000)	1	0.922	0.833	1
Tahta (2019) (Tahta et al., 2019)	0.92	0.864 (2347)	0.792 (76.7)	0.98	0.764	0.903
Riccio (2018) (Riccio et al., 2018)	0.85	0.833 (3000)	–	0.969	0.895	–
Renz (2018) (Renz et al., 2018)	0.52	0.85 (3000)^2^	–	1	0.95	–
Balato (2018) (Balato et al., 2018)	0.875	0.75 (3000)^2^	0.75 (80)^2^	0.971	0.941	0.97
Berger (2017) (Berger et al., 2017)	0.971	0.893 (3000)^2^	0.893 (80)^2^	0.969	0.962	0.922
Sigmund (2019) (Sigmund et al., 2019)	0.69	0.7903 (3000)^3^		0.944	0.94	
Bingham (2014) (Bingham et al., 2014)	1	0.95 (1700)	–	0.95	0.85	–
Median (range)^4^	0.85 (0.52–0.971)	0.864 (0.75–0.902)	0.842 (0.75–0.895)	0.969 (0.922–1)	0.94 (0.764–0.989)	0.949 (0.903–1)

S, sensitivity. Sp, specificity. AD-LF, alpha-defensin lateral flow. LC, leukocyte count. PMN, polymorphonuclear neutrophils. %, percentage. ^1^ The cutoff for the LC was 3000 cells µ L^−1^ in total hip arthroplasties (THAs) and 1700 cells µ L^−1^ in total knee arthroplasties (TKAs). The cutoff for PMN was 80 % in THAs and 65 % in TKAs. ^2^ For the analysis, we have included the cohort of late PJI (Renz) or chronic PJI (Balato and Berger). ^3^ The cutoff for LC was > 10 000 cells µ L^−1^ for acute PJI and > 3000 cells µ L^−1^ for chronic PJI. Data in acute and chronic PJI were aggregated, so it is not possible to split the information. ^4^ Considering only articles using LC cutoff of 3000 cells µ L^−1^ and a PMN of 80 %. Consideration: using an LC cutoff of 3000 cells µ L^−1^ and a PMN of 80 %, AD-LF showed similar sensitivity and specificity compared to both LC and PMN %.

### 3.3 Quality assessment

**Figure 4:**
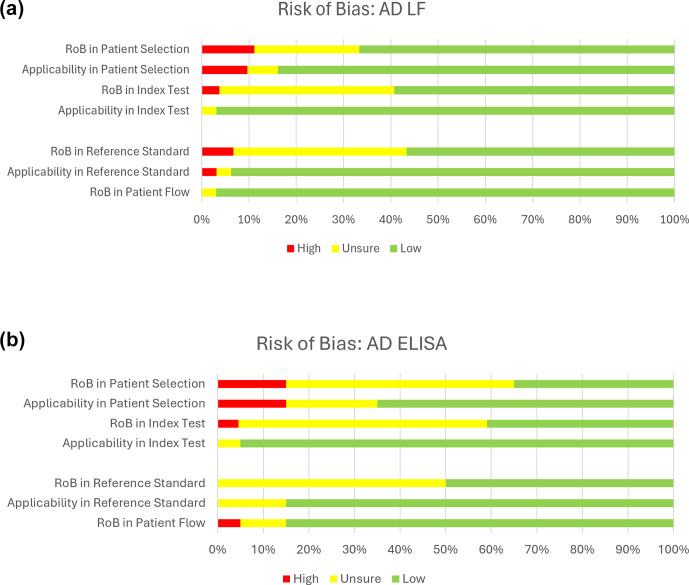
**(a)** Risk of bias for alpha-defensin lateral flow assay (AD-LF). Based on QUADAS-2 assessment. **(b)** Risk of bias for alpha-defensin ELISA assay (AD-LF). Based on QUADAS-2 assessment.

**Figure 5:**
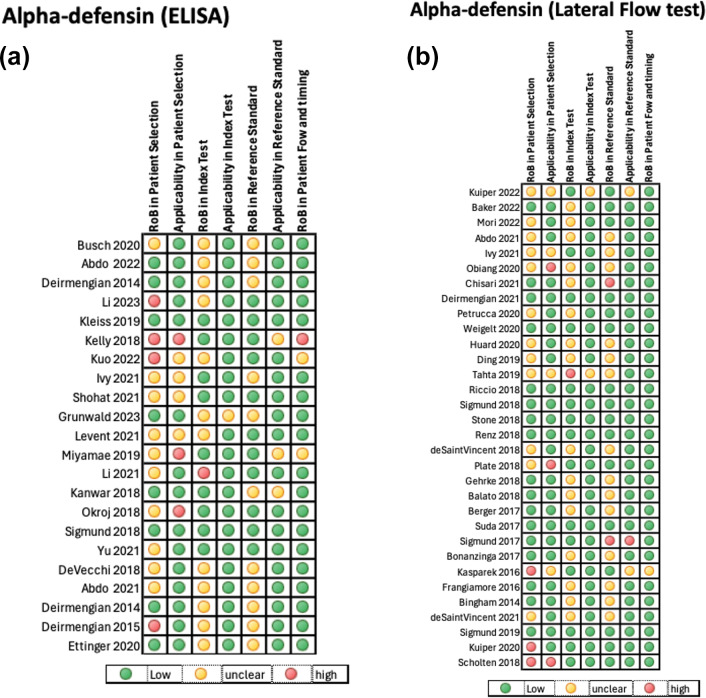
**(a)** QUADAS-2 summary of risk of bias for individual studies evaluating alpha-defensin ELISA assay. **(b)** QUADAS-2 summary of risk of bias for individual studies evaluating alpha-defensin LF test.

The quality of the included studies assessing the diagnostic accuracy of the AD-ELISA and LF assay was evaluated using the QUADAS-2 tool. A pooled analysis of each domain is demonstrated in Fig. 4a and b. The AD-ELISA group had a mean of 66 % low risk of bias across all domains, whereas the AD-LF group had a mean of 74 %. Figure 5a and b demonstrate the risk of bias of each individual study included in analysis.

## 4 Discussion

This systematic review and meta-analysis included 51 studies evaluating the diagnostic accuracy of both the laboratory-based AD-ELISA and the quantitative AD-LF in detecting PJI. Both assays demonstrated excellent specificity, with pooled values of 97.9 % for ELISA and 97.0 % for LF, confirming their reliability as rule-in tests. However, ELISA exhibited higher pooled sensitivity than LF (87.8 % vs. 81.8 %), a difference that likely reflects the intrinsic advantages of its quantitative laboratory design. By measuring alpha-defensin concentration through an enzymatic reaction, ELISA can detect low biomarker levels that may be missed by the qualitative, threshold-based LF assay. Consequently, ELISA demonstrates superior sensitivity and may provide greater reproducibility across a broader range of clinical presentations, including low-grade and indolent infections, while maintaining comparable specificity. Across included studies, heterogeneity was minimal for specificity but substantial for sensitivity, indicating consistent performance in confirming infection but variable reliability in ruling it out. A targeted sub-analysis of studies excluding LC and PMN % from the diagnostic reference standard demonstrated that both AD-ELISA and AD-LF exhibited sensitivity comparable to LC and PMN % but with consistently higher specificity.

There have been several prior systematic reviews and meta-analyses on AD in the diagnosis of PJI (Ahmad et al., 2018; Balato et al., 2018; Carli et al., 2019; Eriksson et al., 2018; Lee et al., 2017; Li et al., 2017; Marson et al., 2018; Saleh et al., 2017; Suen et al., 2018; Vale et al., 2023; Wyatt et al., 2016; Xie et al., 2017; Yuan et al., 2017), with the most recent review published in 2023 (Vale et al., 2023). However, many of these studies employed narrower inclusion criteria, or the meta-analyses have become outdated given the growing body of recent literature on AD in PJI diagnosis. For example, Kuiper et al. (2020b) included only prospective studies in their analysis, while Marson et al. (2018) and Balato et al. (2020) limited inclusion to studies using the MSIS as the diagnostic reference standard. Vale et al. (2023) comprehensively investigated synovial fluid biomarkers across various PJI criteria, reporting favorable diagnostic accuracy for AD consistent with the present study. The present study differs from the previous literature in a few important ways. The present study performed separate pooled analyses for AD-ELISA and AD-LF, allowing more granular comparisons. Additionally, this study incorporated both prospective and retrospective studies and accepted a broader range of validated reference standards, including MSIS 2013, ICM 2018, EBJIS 2021, and IDSA guidelines. Notably, conducting a targeted sub-analysis limited studies that excluded leukocyte count and PMN % from their diagnostic criteria, thereby minimizing incorporation bias and enabling a more direct comparison between AD testing and traditional synovial fluid markers. The methodological breadth, incorporation of the most recent evidence, and strict requirement for accepted gold-standard diagnostic criteria for PJI position our review as one of the most comprehensive evaluations on AD assay performance to date.

A definitive gold standard for diagnosing PJI has yet to be established. Among the available diagnostic tools, synovial fluid analysis has demonstrated the greatest promise for identifying PJI cases with accuracy, outperforming serum markers (Goud et al., 2022). While commonly used, traditional inflammatory markers including white blood cell count (WBC), synovial WBC count, PMN %, and CRP are limited by variable sensitivity, false negatives, and delays in turnaround time (Kheir et al., 2018; Quinlan and Jennings, 2023). Consequently, a growing number of synovial fluid markers have been investigated beyond AD, with notable focus on calprotectin (Peng et al., 2022), interleukin-6 (IL-6) (Li et al., 2022), and leukocyte esterase (Vale et al., 2023), among others. Despite promising early data, many of these alternative biomarkers remain under evaluation, with limited standardization and inconsistent incorporation into consensus guidelines. Further comparative studies are needed to determine the optimal biomarker or combination of markers that can reliably distinguish PJI from aseptic failure across diverse clinical scenarios in TJA.

Quality was assessed using the QUADAS-2 tool, which evaluates risk of bias across four key domains (patient selection, index test, reference standard, and patient flow and timing), along with applicability concerns for each of the first three domains. Overall, the studies included demonstrated low to moderate risk of bias, with the most frequent concerns related to the index test and patient selection domains. Several studies lacked blinding or did not clearly pre-specify thresholds for the index test, which may overestimate diagnostic performance. Nonetheless, the majority adhered to established diagnostic criteria and provided sufficient methodological detail to support inclusion. These findings highlight the importance of cautious interpretation of pooled estimates, especially in the context of heterogeneous study designs and potential incorporation bias.

## 5 Limitations

There are several limitations to this study that should be acknowledged. This meta-analysis and systematic review included significant heterogeneity across the studies included in terms of patient populations, diagnostic criteria, and surgical settings, which may contribute to variability in the reported values. Additionally, confidence intervals were not consistently reported in the included studies, limiting the precision of the pooled estimates of sensitivity and specificity. There is also the potential for publication bias, as studies with favorable results may be more likely to be published. The diagnostic performance of AD may be influenced by implant location due to all total joint arthroplasty being included, chronicity of infection, or underlying inflammatory conditions that were not specifically stratified in our analysis. Finally, a large proportion of the studies included were observational, lacking standardized blinding and clearly defined index test thresholds, introducing the potential for both performance and detection bias. 

## 6 Conclusions

Both AD assays demonstrate high specificity in diagnosing PJI, but AD-ELISA offers superior sensitivity compared to AD-LF and traditional synovial markers. Given variability in the underlying diagnostic criteria for PJI, these results should be interpreted within the context of differing reference standards. These findings support the continued use of AD-ELISA as a valid diagnostic modality. 

## Data Availability

The data used in this study are not publicly available but can be obtained from the corresponding author upon reasonable request.

## Code availability

All analyses were performed using R (R Foundation for Statistical Computing, Vienna, Austria). Analysis scripts and output files are available from the corresponding author upon reasonable request.
